# Evolution of the selfing syndrome in *Ipomoea*

**DOI:** 10.3389/fpls.2013.00301

**Published:** 2013-08-09

**Authors:** Tanya M. Duncan, Mark D. Rausher

**Affiliations:** Department of Biology, Duke UniversityDurham, NC, USA

**Keywords:** *Ipomoea lacunosa*, *Ipomoea cordatotriloba*, selfing syndrome, natural selection, genetic drift

## Abstract

Plants that are highly selfing typically exhibit a suite of morphological traits termed a “selfing syndrome,” including reduced corollas and reproductive structures, loss of corolla pigmentation, little anther-stigma separation, and a lower pollen/ovule (P/O) ratio. While it is typically assumed that these changes are adaptive, few attempts have been made to determine whether they result from the operation of natural selection or genetic drift. In the southeastern United States, *Ipomoea lacunosa* has evolved a typical selfing syndrome compared to its close relative, *Ipomoea cordatotriloba*. Microsatellite markers confirmed that selfing rates are substantially higher in *I. lacunosa*. Furthermore, using a standard Q_ST_ – F_ST_ comparison, we evaluated the relative importance of selection and drift in the evolution of selfing syndrome traits in *I. lacunosa*. The analysis demonstrated that natural selection is responsible for the evolution of reduced corolla size, anther-stigma distance, and style length in this species. By contrast, leaf characteristics unrelated to selfing were found to have diverged largely by genetic drift. Our study provides one of the first confirmations that natural selection drives the evolution of selfing-syndrome traits.

## Introduction

The evolutionary transition from outbreeding to selfing is one of the most common changes in angiosperms, with an estimated 20% of all flowering plants having evolved autogamy as the predominant mode of reproduction (Barrett, 2002). Autogamous plants are typically characterized by a “selfing syndrome,” which consists of having small, scentless, nectarless, and often white flowers, reduced anther-stigma distance, and a decreased pollen/ovule (P/O) ratio (Ornduff, 1969; Sicard and Lenhard, 2011; Kalisz et al., 2012).

Traditionally, the evolution of increased selfing has been viewed as the result of natural selection, either for reproductive assurance when pollinator availability is low or potential mates are commonly absent (Darwin, 1876; Stebbins, 1950; Baker, 1955), or because of a transmission advantage associated with selfing compared to outcrossing (Fisher, 1941; Holsinger, 1988). However, not all traits associated with the selfing syndrome necessarily contribute directly to increased selfing. In particular, selfing-syndrome characters may be divided into two categories: (1) those targeted by natural selection to increase selfing rate, and (2) those that evolved for other reasons. A likely example of a trait in the first category is anther-stigma distance. In many species, it has been shown that reducing anther-stigma distance increases autogamy and increases selfing rate in nature (Chang and Rausher, 1998; Motten and Stone, 2000; Schueller, 2004; Takebayashi et al., 2006). If selection favors increased selfing, it may operate to decrease anther-stigma distance. An example of the second category is (P/O) ratio, which is typically reduced in highly-selfing species. A reduction in P/O by itself is not likely to increase selfing rates. If anything, it is likely to decrease selfing rates because there is less pollen for self-fertilization. It is thus unlikely to be a trait targeted by selection to increase selfing rates. However, once increased selfing has evolved, decreased P/O ratio may be the result of selection to redirect resources to other fitness-enhancing traits because less pollen is needed for effective self-pollination (Brunet, 1992).

A number of selfing-syndrome characters may fall into either category, depending upon when they evolve relative to category 1 traits. Examples are characters that serve to attract pollinators, such as showy petals, scent, and nectar rewards. On the one hand, reduction in attractiveness may itself increase selfing rates by reducing visitation by pollinators carrying pollen from other plants. If these traits are genetically variable when selection arises for increased selfing, they may become targets of selection and hence be category 1 traits. On the other hand, if reduced attractiveness does not reduce visitation, or if there is little genetic variation in these traits when selection for increased selfing arises, these traits may not evolve in response to such selection. Subsequently, however, other forms of selection, including selection for reducing costs of production, for shortening the time involved in reproduction, or for reducing floral herbivory, may act to alter these characters [reviewed in Sicard and Lenhard (2011)].

These arguments all assume that some form of natural selection molds the suite of characters that comprise the selfing syndrome. Seldom considered is the alternative possibility that some of these characters are neutral and have evolved by genetic drift. Such change, however, is plausible. For example, once a population has become highly selfing, there is no longer a need to attract pollinators, and selection to maintain attractive traits would be relaxed. It is easy to imagine that under these circumstances, loss-of-function mutations that abolish function could accumulate to reduce pigmentation, scent, and nectar production. Similarly, it is also conceivable that drift could frequently lead to a general reduction in flower size, especially if mutations reducing flower size are more frequent than mutations that increase flowers size. The possible importance of genetic drift is enhanced by the fact that selfing tends to reduce genetic diversity within populations. In particular, inbreeding associated with selfing tends to increase homozygosity (Charlesworth and Wright, 2001). Excess homozygosity in turn lowers the effective population size (Ne) and increases the effect of genetic drift in selfing plants (Pollak, 1987).

Our objective in this study was to explicitly examine whether natural selection was responsible for the evolution of selfing-syndrome characters in the annual morning glory *Ipomoea lacunosa*, which has diverged from the closely related *Ipomoea cordatotriloba* in floral morphology (Duncan, 2013, in press). Specifically, *I. lacunosa* has a short anther-stigma distance, small, white flowers, and a low P/O ratio compared to *I. cordatotriloba*, which has a greater anther-stigma distance, larger, purple flowers, and a higher P/O ratio (Austin, 1978; McDonald et al., 2011). We first demonstrate, using microsatellite markers that selfing rate is generally substantially higher for *I. lacunosa* than for *I cordatotriloba*. We then use a Qst-Fst comparison to determine whether two selfing-syndrome traits exhibit divergence that is inconsistent with the operation of genetic drift. One character is reduced anther-stigma distance, which we expected to have been favored by selection because it is a character that presumably affects selfing rate substantially. The second character we examine is flower size, a presumably attractive trait, which is reduced in *I. lacunosa*. For this trait, we had no a priori expectation about the involvement of natural selection.

## Materials and methods

### Study system

*I. lacunosa* and *I. cordatotriloba* are noxious weeds that are indigenous to the southeastern United States (Jones and Deonier, 1965). The two plants have different floral morphologies, with *I. lacunosa* typically having small white flowers, and *I. cordatotriloba* normally having larger, purple flowers (Abel and Austin, 1981). A recent analysis of shared genetic variation suggests that *I. lacunosa* and *I. cordatotriloba* are exchanging genetic material because they are genetically indistinguishable at neutral markers (Duncan, 2013, in press). Reflecting this genetic similarity, the two species can cross and produce viable offspring (Abel and Austin, 1981; Duncan, 2013, in press). In North Carolina (NC) and South Carolina (SC) the ranges of the two species overlap, with *I. lacunosa* growing along the coast as well as in the central area of the two states, while *I. cordatotriloba* is found predominately along the coast (Duncan, 2013, in press). In NC and SC the plants germinate in late May and begin to flower in August or early September. Flowering ceases sometime in mid to late fall and plants die at the first hard frost. Plants of each taxon are self-compatible; however, *I. cordatotriloba* has been described as having a mixed mating system (selfing and outcrossing), while *I. lacunosa* is thought to reproduce largely by self-pollination (McDonald et al., 2011).

#### Geographic survey and floral measurements

**Figure 1:**
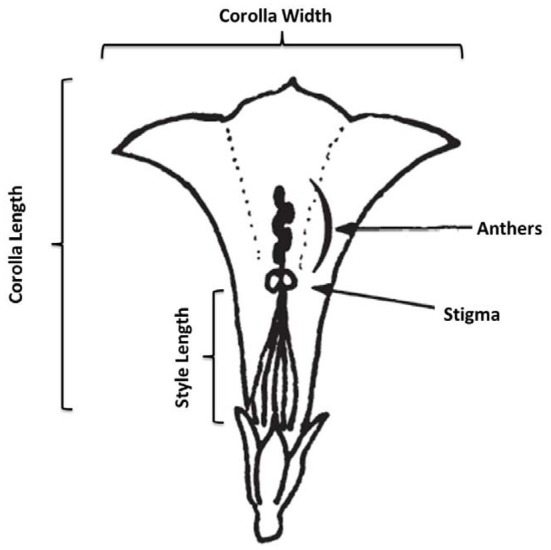
**Schematic of corolla measurements.** In the figure anthers and stigma are not touching (anther-stigma distance = 1). Figure adapted from Abel and Austin (1981).

In August and September of 2010, we conducted a census of 7 populations of *I. cordatotriloba* and five populations of *I. lacunosa* in NC and SC. In each population we measured morphological traits on an average of 12 and 20 individuals, respectively, for the two species. During the survey morphological measurements of flower width, flower length, flower width-length ratio, anther-stigma separation, style length, leaf length, leaf width, and leaf width-length ratio (Figure 1). Anther-stigma distance was quantified as 0 if all anthers touched the stigma, 0.5 if at least one, but no more than 4, touched the stigma, and as 1 if no anthers touched the stigma. Correlations among traits within a population were calculated using JMP®, Version 9, SAS Institute Inc., Cary, NC, 1989–2007. The average correlation coefficient for all populations was then calculated.

#### Microsatellite scoring

During the survey, leaf tissue was collected for genotyping microsatellite markers from each measured plant. DNA was extracted using a CTAB protocol (Doyle and Doyle, 1981). We used primers that had been developed for *I. trifidia* but were also reported to amplify microsatellite regions in *I. lacunosa* (Hu et al., 2004). Out of the eight microsatellites reported to amplify in *I. lacunosa*, we found that only four amplified and contained sufficient variability to distinguish among the study taxa. Each of the four microsatellite markers was amplified with Hex or Fam fluorescently labeled primers, using KAPA taq (Kapa Biosystems, Woburn, Massachusetts, USA), and fragment analysis was conducted on a ABI 3730 × 1 DNA Analyzer. Each marker was visually scored using the software GENEMARKER (SoftGenetics, 2005, State College, Pennsylvania, USA).

### Estimating selfing rates

We did not estimate selfing rates directly; instead, selfing rates were estimated indirectly from neutral markers (microsatellites). In particular, our sampling of populations and individuals allowed us to estimate selfing rates from observed heterozygosity, *H*. Selfing rate is related the inbreeding coefficient, *F*, by

s = 2 F/(1 + F)

(Hartl and Clark, 1997). In turn, the inbreeding coefficient is related to heterozygosity by

$$F=(H_{0}-H)/H_{0}$$

where *H*_0_ is the expected heterozygosity based on allele frequencies and random mating (Hartl and Clark, 1997). Combining these two equations yields the relationship between selfing rate and heterozygosity:

$$s=2(H_{0}-H)/(2H_{0}-H)$$

Estimation of selfing rates for individual populations were based on four microsatellite loci sampled from 97 *I. cordatotriloba* individuals from 7 populations and 110 *I. lacunosa* individuals from 8 populations (Duncan, 2013, in press). Observed and expected heterozygosity were calculated using the program Arlequin 3.5 (Excoffier and Lischer, 2010).

### Relationship between morphological variables and selfing rate in *I. cordatotriloba*

The statistical significance of variation in morphological characters among *I. cordatotriloba* populations was assessed using a one-way analysis of variance as implemented by the GLM procedure in SAS (SAS Institute Inc., Cary, NC, 1989–2007). The relationship between population means for selfing rate and morphological variables and their squares was assessed by regression analysis using a backward elimination procedure implemented by the REG procedure in SAS. The critical value for keeping a variable in the model was set to *p* = 0.05.

### Morphological trait divergence

To determine whether trait divergence between the two species is larger than expected under neutrality, we used a modified F_ST_ – Q_ST_ approach (Whitlock and Gilbert, 2012). The standard F_ST_ – Q_ST_ approach compares populations at a single spatial level (e.g., Leinonen et al., 2008; Whitlock, 2008). However, in our analysis populations are nested within species and we are primarily concerned with examining divergence between species. There are thus two hierarchical “spatial” levels.

For each morphological trait measured, we calculated the summary statistic *Q*_CT_ using the method developed by Whitlock and Gilbert (Whitlock and Gilbert, 2012). *Q*_CT_ describes the magnitude of variation for a morphological trait between *I. lacunosa* and *I. cordatotriloba*. Under the assumption that trait divergence is due to genetic drift, the expected value of *Q*_CT_ is

$$Q_{CT}=V_{C}/(V_{C}+V_{P}+2V_{I})=F_{CT}$$

where *V*_*C*_, *V*_*P*_, and *V*_*I*_ are the components of genetic variance among species, among populations, and among individuals within populations, respectively (Whitlock and Gilbert, 2012) and *F*_CT_ is the between-species *F*-statistic for the microsatellite loci.

To test whether natural selection contributed to trait divergence between the two species, we tested whether the null hypothesis of no selection, corresponding to the above equation, could be rejected in favor of the hypothesis that *Q*_CT_ > *F*_CT_. Using bootstrap sampling, we compared the distributions of *Q*_CT_ and *F*_CT_. To generate a bootstrap sample from one of the species, we first randomly chose a population from that species. We then drew *n* individuals randomly with replacement from those in that population, where *n* was the actual number of individuals scored in that population. We continued sampling in this way until the number of populations in the sample was equal to the number actually scored. For each species, we preformed 1000 bootstrap samples.

For each bootstrap sample, we calculated *Q*_CT_ or *F*_CT_ from the variance components from a standard nested ANOVA. Because *V*_*I*_ is the additive genetic variance within populations and could not be calculated for the traits from our data, we instead examined two extremes: heritability = 1 and heritability = 0. For heritability = 1, *V*_*I*_ was set equal to the within-population component of variation from the nested ANOVA for the trait (equal to the within-population phenotypic variance); for heritability = 0, we set *V*_*I*_ = 0. Because both approaches led to similar results, we report only the results for *V*_*I*_ = within population variance component. This approach is conservative because it produces a smaller *Q*_CT_.

## Results

### Selfing rates

The eight populations of *I. lacunosa* we sampled uniformly exhibited very high selfing rates (mean *s* = 0.955 ± 0.018 Table 1). By contrast, *I. cordatotriloba* populations were more variable across populations [mean *s* = 0.511 ± 0.182 (negative estimates considered 0); Table 1]. This difference is statistically significant (one-tailed *t*-test: *t* = 2.43, *df* = 13, *p* < 0.025). It thus appears that selfing rate is on average substantially higher for *I. lacunosa* than for *I. cordatotriloba*.

**Table 1:** **Measures of genetic diversity and selfing rate for 7 populations of *I. cordatotriloba* and 8 populations of *I. lacunosa* from North Carolina and South Carolina**.

**Population**	***N***	***NPL***	***A***	***Ho***	***He***	***s***
***I. CORDATOTRILOBA***						
cle1	18	2	2.0 (0.0)	0.03	0.33	0.95
c7	13	4	3.0 (1.4)	0.61	0.55	−0.24
c13	23	2	2.5 (0.7)	0.04	0.27	0.92
cl4	12	3	2.7 (1.2)	0.06	0.43	0.93
c22	8	4	2.5 (0.6)	0.50	0.43	−0.39
c29	6	1	2.0 (0.0)	0.30	0.30	0.00
clela3	17	2	2.0 (0.0)	0.18	0.50	0.78
***I. LACUNOSE***						
la3	14	1	2.0 (0.0)	0.00	0.50	1.00
la15	14	2	4.0 (0.0)	0.04	0.37	0.94
lela8	31	2	2.5 (0.7)	0.05	0.26	0.89
la43	8	2	2.0 (0.0)	0.00	0.38	1.00
la30	14	2	2.0 (0.0)	0.04	0.20	0.89
la35	16	2	3.0 (1.4)	0.03	0.20	0.92
la7	6	1	2.0 (0.0)	0.00	0.30	1.00
ula7	6	1	3.0 (0.0)	0.00	0.55	1.00

*N, sample size; NPL, number of polymorphic loci; A, mean (sd) number of alleles; Ho, observed heterozygosity; He, expected heterozygosity; s, selfing rate*.

**Figure 2:**
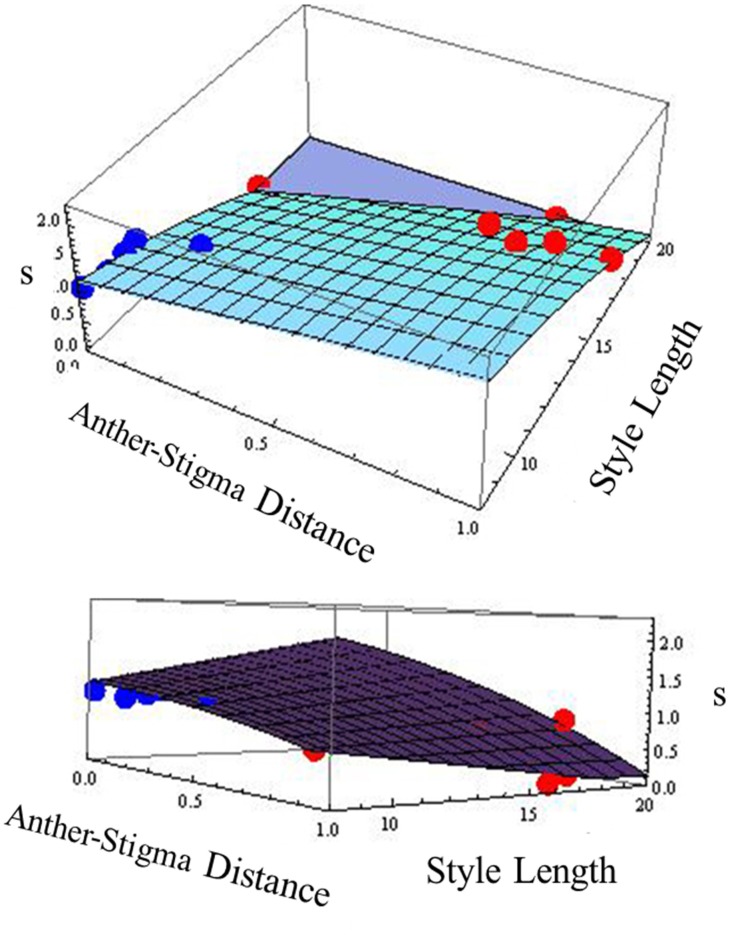
**Relationship across populations between selfing rate (s) and anther-stigma distance and style length.** The best-fit surface is specified by *s* = 1.482 + 0.818 asd −0.0054 style length^2^ [*R*^2^ = 0.79, *F*_(2, 9)_ = 16.58, *p* < 0.001]; Significance of asd: *F*_(1, 9)_ = 11.63, *p* = 0.0077. Significance of style length^2^: *F*_(1, 9)_ = 31.36, *p* = 0.0003. Red points: *I. cordatotriloba*. Blue points: *I. lacunosa*. The two figures are different views of the same relationship.

Because in many plant species, variation in selfing rate is associated with variation in floral morphological characteristics, we examined the relationship between selfing rate and morphological variables corolla length and width, anther-stigma distance and style length and their squares for the combined data from the two species (Table 2). In a backward-elimination multiple regression involving these variables, all variables except anther-stigma distance and the square of style length were eliminated from the regression. The regression involving these two variables explained 79% of the variation in selfing rate (Figure 2). Anther-stigma distance and style length were both highly significant [*F*_(1, 9)_ = 11.63 and 31.36, *p* < 0.0077 and 0.0003, respectively]. Moreover, each of these variables differed significantly between the two species [Anther-stigma distance: *F*_(1, 10)_ = 20.14, *p* = 0.0012; Square of style length: *F*_(1, 10)_ = 64.93, *p* < 0.0001]. It thus appears that much of the variation between species is due to the joint effects of these two morphological variables.

**Table 2:** **Mean ± SD for morphological measurements for populations of *I. cordatotriloba* and *I. lacunosa* with concordant microsatellite data**.

	**Population**	**Species**	***n***	**Corolla length (mm)**	**Corolla width (mm)**	**Corolla L/W ratio**	**Anther stigma distance**	**Style length (mm)**	**Leaf length (mm)**	**Leaf width (mm)**	**Leaf L/W ratio**
1	cle1	*I. cordatotriloba*	10	25.50 ± 1.96	29.00 ± 1.18	0.88 ± 0.06	1.00 ± 0.00	16.20 ± 0.75	41.40 ± 10.97	43.60 ± 17.28	1.01 ± 0.18
2	c7	*I. cordatotriloba*	5	21.60 ± 0.49	29.80 ± 10.33	0.73 ± 0.03	0.00 ± 0.00	16.80 ± 0.75	25.20 ± 6.52	24.20 ± 6.52	1.10 ± 0.25
3	c13	*I. cordatotriloba*	20	21.60 ± 1.81	28.23 ± 2.99	0.77 ± 0.08	0.78 ± 0.40	15.74 ± 1.56	41.73 ± 10.64	37.68 ± 10.68	1.13 ± 0.20
4	cl4	*I. cordatotriloba*	14	26.71 ± 2.46	32.57 ± 2.44	0.83 ± 0.10	0.86 ± 0.34	16.21 ± 1.11	50.07 ± 9.69	38.79 ± 8.78	1.30 ± 0.13
5	c22	*I. cordatotriloba*	6	27.57 ± 2.04	32.07 ± 2.06	0.86 ± 0.08	0.86 ± 0.23	17.50 ± 1.16	39.71 ± 4.27	39.00 ± 7.31	1.04 ± 0.11
6	clela3	*I. cordatotriloba*	20	24.90 ± 2.78	30.65 ± 2.46	0.81 ± 0.08	0.68 ± 0.40	16.55 ± 1.0	53.33 ± 14.28	46.03 ± 11.77	1.18 ± 0.26
7	c29	*I. cordatotriloba*	8	32.63 ± 1.88	36.63 ± 2.06	0.89 ± 0.07	0.75 ± 0.43	19.88 ± 0.93	52.44 ± 11.94	47.94 ± 12.42	1.13 ± 0.24
8	la3	*I. lacunosa*	19	15.44 ± 1.21	12.94 ± 1.03	1.20 ± 0.09	0.00 ± 0.00	8.17 ± 0.50	48.17 ± 9.31	37.50 ± 8.67	1.30 ± 0.10
9	la15	*I. lacunosa*	20	17.48 ± 1.12	14.85 ± 1.19	1.18 ± 0.08	0.00 ± 0.00	10.02 ± 0.53	31.65 ± 11.93	24.45 ± 11.35	1.38 ± 0.24
10	lela8	*I. lacunosa*	16	15.56 ± 1.94	15.00 ± 1.70	1.04 ± 0.11	0.09 ± 0.21	9.25 ± 0.66	34.72 ± 11.73	28.22 ± 10.73	1.30 ± 0.28
11	la43	*I. lacunosa*	29	18.14 ± 1.88	16.17 ± 1.34	1.23 ± 0.14	0.00 ± 0.00	10.54 ± 1.08	56.86 ± 25.42	43.76 ± 20.87	1.42 ± 0.72
12	ula7	*I. lacunosa*	18	18.34 ± 1.70	16.68 ± 1.75	1.11 ± 0.10	0.15 ± 0.23	11.16 ± 1.18	35.11 ± 14.16	26.10 ± 12.18	1.38 ± 0.21

As remarked above, *I. cordatotriloba* populations are substantially variable for selfing rate. Among just these populations, a multiple regression of selfing rate on anther-stigma distance and the square of style length yielded a significant effect of the latter variable [*F*_(1, 4)_ = 8.40, *p* = 0.0442]. The effect of anther-stigma distance was not quite significant [*F*_(1, 4)_ = 1.98, *p* = 0.118], though this is perhaps not surprising given the small number of populations. Overall, the square of style length explains 52% of the variation in selfing rate among *I. cordatotriloba* populations and anther-stigma distance explains another 27%. Moreover, the estimated regression coefficients are similar for *I. cordatotriloba* and the combined 2-species sample (anther-stigma distance: 0.72 ± 0.361 vs. 0.81 ± 0.24, respectively; square of style length: −0.0070 ± 0.0024 vs. −0.0053 ± 0.00096, respectively). It thus seems likely that, as for the overall difference in selfing rates between the two species, these variables also account for much of the variation in selfing rate among *I. cordatotriloba* populations.

### Selection on divergent traits

To test whether divergence in floral morphological traits between *I. cordatotriloba* and *I. lacunosa* is consistent with neutral divergence, we conducted a hierarchal *Q*_CT_ − *F*_CT_ analysis. If *Q*_CT_ > *F*_CT_, divergence in the trait is typically inferred to be caused by selection. By contrast, if *Q*_CT_ = *F*_CT_, then the data is consistent with divergence by genetic drift. We performed a comparison of *Q*_CT_ vs. *F*_CT_ for the measured floral traits, as well as for leaf traits that served as a control.

**Figure 3:**
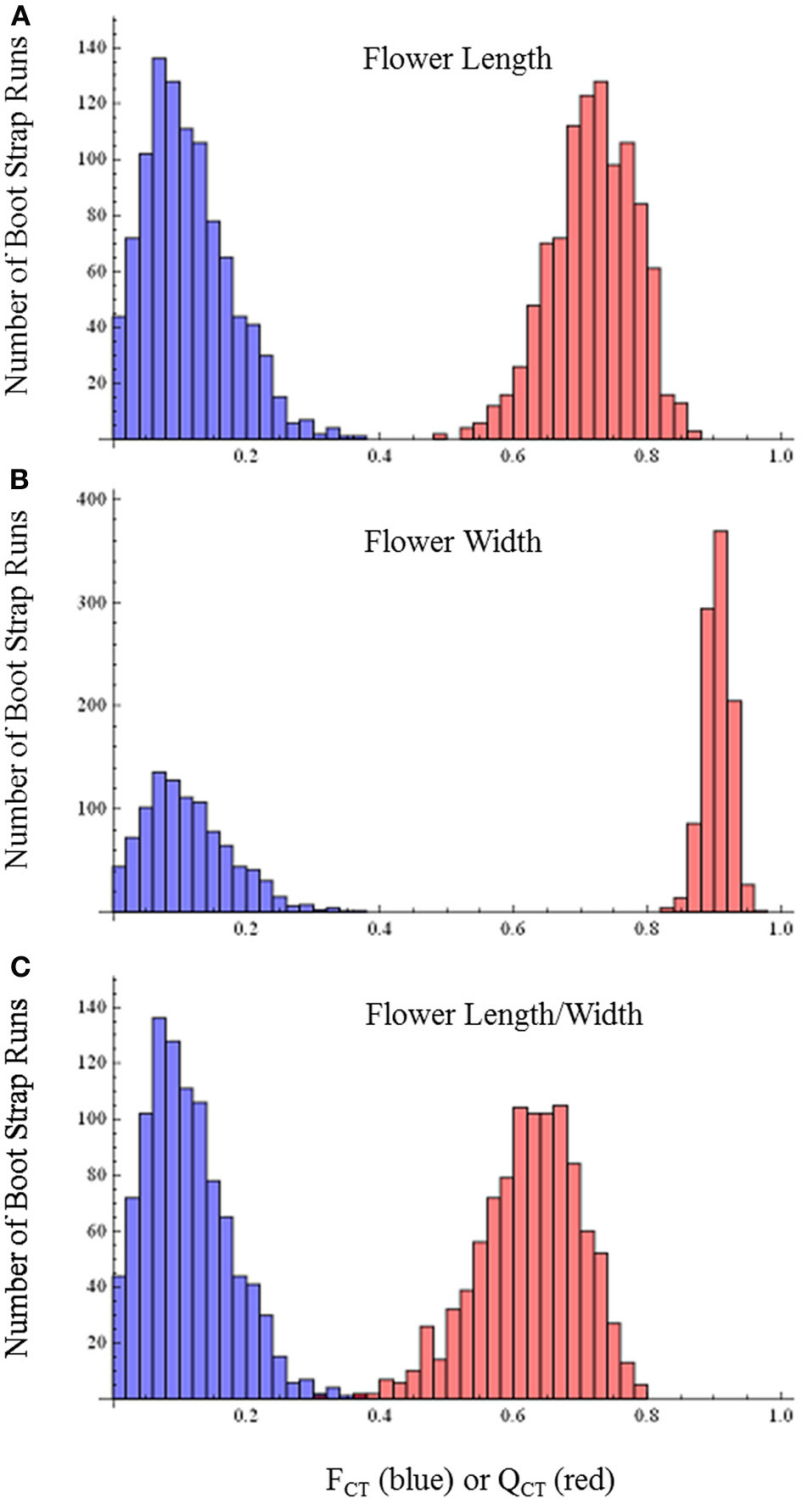
***F*_CT_ vs. *Q*_CT_ analyses (A) flower length (*p* < 0.001) (B) flower width (*p* < 0.001) (C) flower length-width ratio (*p* < 0.001)**.

**Figure 4:**
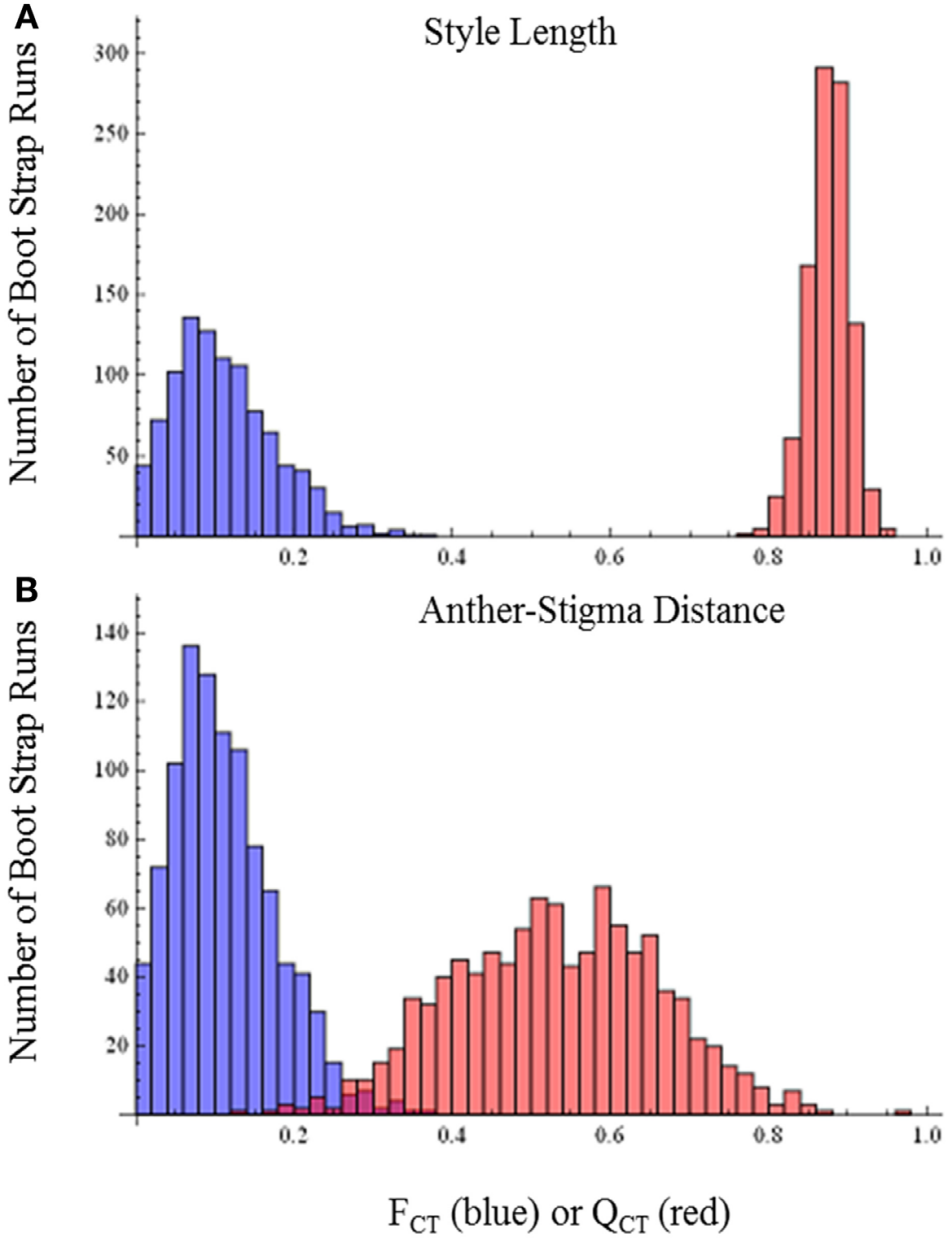
***F*_CT_ vs. *Q*_CT_ analyses (A) style length (*p* < 0.001) (B) anther-stigma distance (*p* = 0.002)**.

*Q*_CT_ values calculated for flower length, flower width, and flower length-width ratio are extremely differentiated from *F*_CT_ values (Figure 3). For flower length and width, the distribution of bootstrap values of *F*_CT_ does not overlap the distribution for *Q*_CT_, while for the ratio of floral length/width, there is only minimal overlap. In all three cases the significance of the difference is *p* < 0.001, indicating divergence is inconsistent with neutral expectations. Because these traits are moderately correlated (Table 3), these tests may not be independent and divergence in these traits may reflect selection on a single, composite developmental character. Style length and anther-stigma distance are less correlated with corolla length and width and with each other than are corolla length and width to each other (Table 3). Thus, style length and anther-stigma distance presumably represent separate developmental modules, while corolla length and width do not. However, for these characters there is little or no overlap between the bootstrap distributions of *Q*_CT_ and *F*_CT_ (Figure 4). Both of these differences are significant (style length, *p* < 0.001; anther-stigma distance, *p* = 0.002), again indicating inconsistency with neutral expectations.

**Table 3:** **The average phenotypic correlations ± standard error between pairs of measured traits within populations of *I. cordatotriloba* and *I. lacunosa***.

	**Corolla length**	**Corolla width**	**Corolla length/width**	**Anther-stigma distance**	**Style length**	**Leaf length**	**Leaf width**
Corolla length							
Corolla width	0.36 ± 0.23						
Corolla length/width	0.56 ± 0.20	−0.43 ± 0.36					
Anther-stigma distance	−0.03 ± 0.16	−0.03 ± 0.29	0.00 ± 0.23				
Style length	0.20 ± 0.33	0.28 ± 0.25	0.04 ± 0.33	0.07 ± 0.19			
Leaf length	−0.02 ± 0.28	0.08 ± 0.36	−0.10 ± 0.38	0.08 ± 0.28	−0.04 ± 0.35		
Leaf width	−0.04 ± 0.32	0.17 ± 0.28	−0.15 ± 0.23	0.07 ± 0.31	−0.10 ± 0.44	0.79 ± 0.22	
Leaf length/width	−0.10 ± 0.20	−0.05 ± 0.36	0.04 ± 0.26	0.00 ± 0.23	0.12 ± 0.36	−0.06 ± 0.38	0.43 ± 0.43

**Figure 5:**
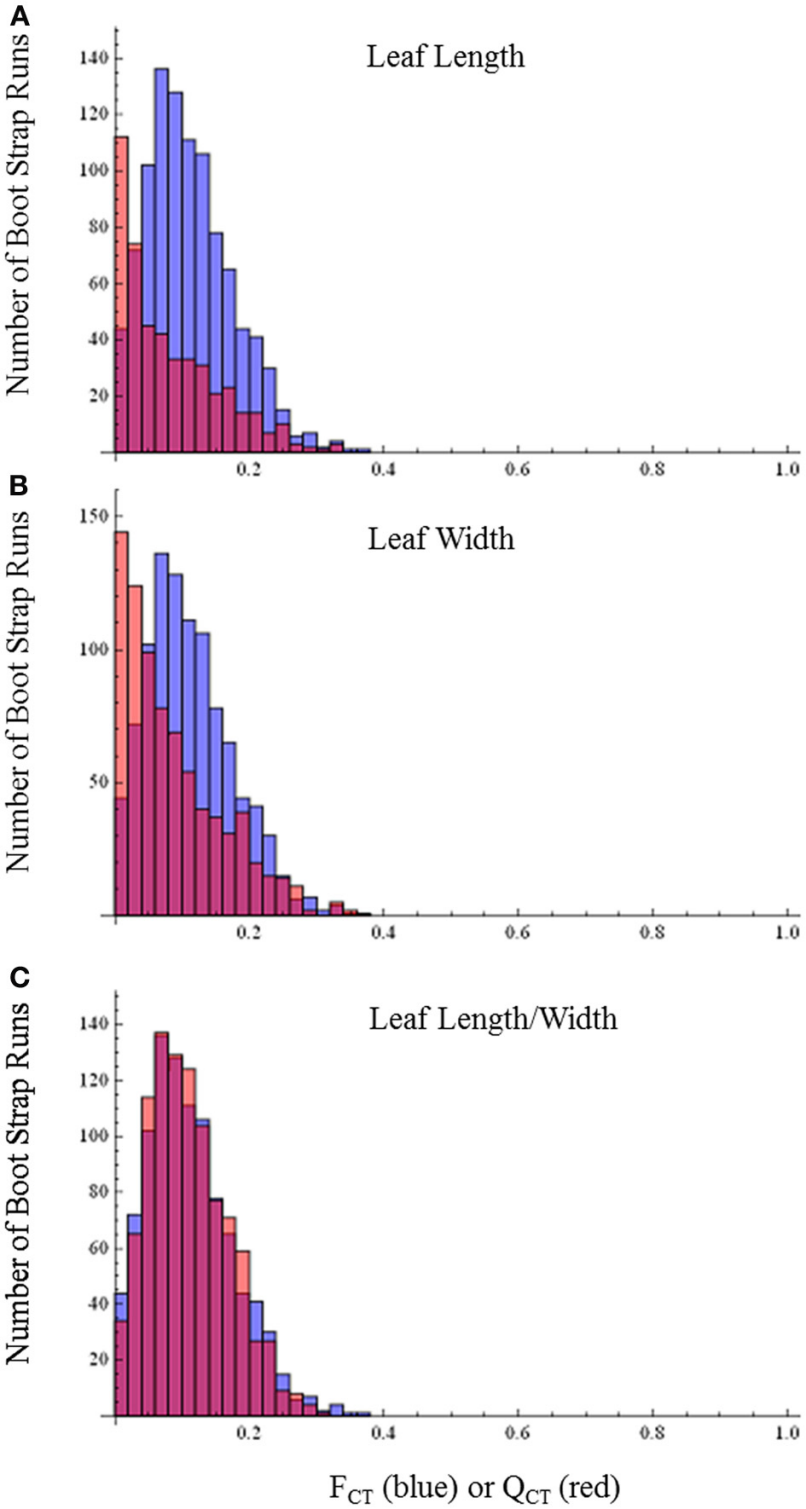
***F*_CT_ vs. *Q*_CT_ analyses (A) leaf length (*p* = 0.80) (B) leaf width (*p* = 0.64) (C) leaf length-width ratio (*p* = 0.44).** Orange bars indicate portion of red bar that does not overlap blue bar.

By contrast with the floral characters, distributions of *Q*_CT_and *F*_CT_ values broadly overlap for leaf length, leaf width, leaf length-width ratio (Figure 5) and differences are not statistically significant (leaf length, *p* = 0.80; leaf width, *p* = 0.64; leaf length-width ratio, *p* = 0.44). This pattern provides no evidence for selection causing divergence in these traits and indicates they are diverging neutrally.

## Discussion

### Evolution of selfing rates

Our results verify that *I. lacunosa* is highly selfing whereas *I. cordatotriloba* appears to have a mixed mating system. This difference in selfing rates is explainable by reduced anther-stigma distance in *I. lacunosa*, which in other plant species increases autogamy (Chang and Rausher, 1998; Schueller, 2004; Takebayashi et al., 2006), as well as by a reduction in style length.

*I. cordatotriloba* populations are much more variable in estimated selfing rate than *I. lacunosa* populations. Some populations exhibit selfing rates nearly as high as those in *I. lacunosa*, whereas others are estimated to be largely outcrossing (Table 1). Although spatial variation in the availability of pollinators could conceivably contribute to this variation (Barrett et al., 1996), most of it appears to be explainable by variation in floral morphology. In particular, selfing rate seems to be determined largely by anther-stigma distance and style length. While reduced anther-stigma distance typically increases selfing rates in plants (Motten and Stone, 2000), it is unclear how reduced style length contributes to increased selfing in the two *Ipomoea* species. The existence of between-population variation in these characters in *I. cordatotriloba* suggests that standing genetic variation in these characters may have facilitated even further reduction in anther-stigma distance and style length in *I. lacunosa*.

### Selection on category 1 traits

As described in the introduction, Category 1 traits are traits for which variation directly affects selfing rate and that are likely targets of selection acting to alter selfing rate. Our analyses suggest that both anther-stigma distance and also style length are likely Category 1 traits. Both of these traits are highly correlated with selfing rates at the population level.

Our *a priori* expectation was that divergence in Category 1 traits would likely be caused by natural selection rather than genetic drift because it is difficult to imagine that variation in selfing rate would be selectively neutral. In order to be neutral, there would have to be precise tradeoffs among inbreeding depression, pollen discounting, and the benefits of reproductive assurance (Holsinger, 1988) that yielded no fitness differences among genotypes with different character values of these traits, which would require an improbable balance of effects. This expectation was in fact realized: both anther-stigma distance and style length exhibited a *Q*_CT_ between species that was substantially greater than *F*_CT_, indicating that divergence is inconsistent with neutral processes.

Although a number of advantages have been suggested for increased selfing, including pollinator uncertainty, avoidance of interspecific hybridization, and increased genetic transmission (Fisher, 1941; Smith and Rausher, 2007), we currently have no information that would allow us to distinguish among these possibilities. However, it is likely that the evolution of high levels of selfing set the stage for the subsequent evolution of other selfing-syndrome characters.

### Selection on other traits

Our results indicate that divergence between *I. lacunosa* and *I. cordatotriloba* in floral size was driven by natural selection. One possible explanation for this divergence is that it is the result of indirect selection due to correlations between anther-stigma distance and corolla dimensions and selection on the former. We believe, however, that this explanation is unlikely. Although we did not measure genetic correlations among these traits, the phenotypic correlations between anther-stigma distance and corolla dimensions are very low and not significantly different from 0. We suspect these reflect underlying low genetic correlations that would essentially preclude the evolution of reduced corollas due to indirect selection.

It also seems unlikely that direct selection to reduce selfing rates acted to reduce corolla size in *I. lacunosa*. Because variation in anther-stigma distance and style length accounts for approximately 80% of the variation in selfing rates, while corolla dimensions are not significantly related to selfing rate, there is little scope for this type of selection. Instead, we suspect that reduction in corolla size in *I. lacunosa* resulted from selection to redirect resources from petals to other fitness-enhancing traits (Brunet, 1992). Final resolution of this issue, however, must await functional analysis of selection on these traits.

### Selection on vegetative traits

Even though floral size and mating systems have been found to be extremely correlated, a systematic survey of outcrossing and selfing species found that there is a much weaker correlation between flower size and vegetative tissue dimensions, such as leaf length or width (Ashman and Majetic, 2006). We also found very weak phenotypic correlations between floral traits and leaf size, suggesting that evolution of leaf traits should not be influenced by indirect selection on floral traits. Our results are consistent with this expectation. In particular, we found no significant differences between *F*_CT_ and *Q*_CT_ for leaf dimensions, indicating that divergence in leaf traits are primarily due to genetic drift. Thus, as has been found in other systems (Ashman and Majetic, 2006), evolution of leaf size appears to be independent of the evolution of floral characters.

### Conflict of interest statement

The authors declare that the research was conducted in the absence of any commercial or financial relationships that could be construed as a potential conflict of interest.
